# TrimetaziDine as a Performance-enhancING drug in heart failure with preserved ejection fraction (DoPING-HFpEF): rationale and design of a placebo-controlled cross-over intervention study

**DOI:** 10.1007/s12471-020-01407-z

**Published:** 2020-03-11

**Authors:** A. A. van de Bovenkamp, A. J. Bakermans, C. P. Allaart, A. J. Nederveen, W. E. M. Kok, A. C. van Rossum, M. L. Handoko

**Affiliations:** 1 Department of Cardiology, Amsterdam UMC, Vrije Universiteit Amsterdam, Amsterdam Cardiovascular Sciences, Amsterdam, The Netherlands; 2 Department of Radiology and Nuclear Medicine, Amsterdam UMC, University of Amsterdam, Amsterdam, The Netherlands; 3 Department of Clinical and Experimental Cardiology, Amsterdam UMC, University of Amsterdam, Amsterdam Cardiovascular Sciences, Heart Center, Amsterdam, The Netherlands

**Keywords:** Heart failure, diastolic, Trimetazidine, Catheterisation, Swan-Ganz, Pulmonary wedge pressure, Exercise, Magnetic resonance spectroscopy

## Abstract

**Background:**

Currently, no specific treatment exists for heart failure with preserved ejection fraction (HFpEF). Left ventricular (LV) relaxation during diastole is a highly energy-demanding process, while energy homeostasis is known to be compromised in HFpEF. We hypothesise that trimetazidine – a fatty acid β‑oxidation inhibitor – improves LV diastolic function in HFpEF, by altering myocardial substrate use and improving the myocardial energy status.

**Objectives:**

To assess whether trimetazidine improves LV diastolic function by improving myocardial energy metabolism in HFpEF.

**Methods:**

The DoPING-HFpEF trial is a randomised, double-blind, placebo-controlled cross-over intervention trial comparing the efficacy of trimetazidine and placebo in 25 patients with stable HFpEF. The main inclusion criteria are: New York Heart Association functional class II to IV, LV ejection fraction ≥50%, and evidence of LV diastolic dysfunction. Patients are treated with one 20-mg trimetazidine tablet or placebo thrice daily (twice daily in the case of moderate renal dysfunction) for two periods of 3 months separated by a 2-week washout period. The primary endpoint is the change in pulmonary capillary wedge pressure during different intensities of exercise measured by right heart catheterisation. Our key secondary endpoint is the myocardial phosphocreatine (PCr)/ATP ratio measured by phosphorus-31 magnetic resonance spectroscopy and its relation to the primary endpoint. Exploratory endpoints are 6‑min walk distance, *N*-terminal pro-brain natriuretic peptide levels, and quality of life.

**Conclusion:**

The DoPING-HFpEF is a phase-II trial that evaluates the effect of trimetazidine, a metabolic modulator, on diastolic function and myocardial energy status in HFpEF. [EU Clinical Trial Register: 2018-002170-52; NTR registration: NL7830]

## Background

**Fig. 1:**
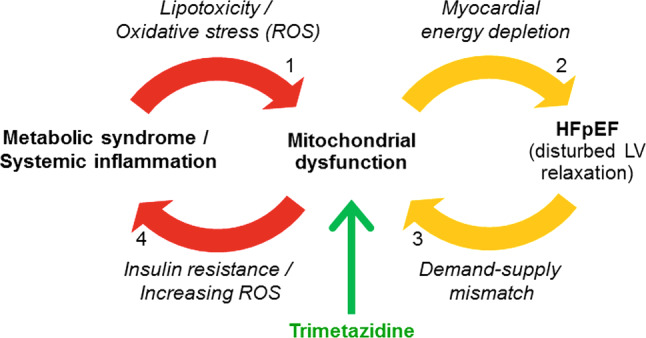
Proposed relationship between heart failure with preserved ejection fraction (*HFpEF*), mitochondrial dysfunction and metabolic syndrome, and the therapeutic potential of trimetazidine. The numbers of the arrows correspond to those in the main text. *LV* left ventricular

Heart failure with preserved ejection fraction (HFpEF) is a growing healthcare burden and its prevalence is increasing: it currently accounts for approximately half of all new heart failure cases [1]. Similar to heart failure with a reduced ejection fraction (HFrEF), the prognosis of HFpEF is grim, but unlike HFrEF no specific therapies exist thus far [2]. Patients complain of exertional dyspnoea, which is pathophysiologically linked to left ventricular (LV) diastolic dysfunction with high filling pressures, although other factors contribute as well [3]. Recently, this heart failure phenotype has been specifically related to obesity and the metabolic syndrome [4]. Mechanistic links between HFpEF and the metabolic syndrome currently focus on systemic inflammation resulting in oxidative stress, with impaired paracrine signalling between endothelial cells and cardiomyocytes by the NO-cGMP-PKG pathway [5]. Unfortunately, trials that have targeted this specific pathway have been unsuccessful [6]. Here, we propose to investigate mitochondrial (dys)function and myocardial energy depletion in HFpEF as a novel link between metabolic syndrome and impaired LV relaxation (Fig. 1).

### HFpEF and the ‘energy depletion’ hypothesis

LV relaxation is a highly energy-demanding process. During diastole, calcium ions are actively transported back into the sarcoplasmic reticulum of the cardiomyocyte by sarcoplasmic/endoplasmic reticulum Ca^2+^-ATPase (SERCA) pumps, and adenosine triphosphate (ATP) is also required for cross-bridge detachment [7]. In the case of ischaemia, defined as insufficient coronary blood flow to the myocardium to meet the metabolic demand, disturbances in myocardial relaxation are among the first events to occur, even before contractility becomes impaired [8]. In HFpEF patients, Phan et al. detected a myocardial energy deficiency that may underlie malfunctioning of the active relaxation stage of diastole, particularly during exercise (Fig. 1, arrow 2) [9]. This is supported by the findings of other studies showing a correlation between reduced myocardial phosphocreatine (PCr)/ATP ratios and the severity of diastolic dysfunction [10, 11]. Interestingly, in asymptomatic patients with type 2 diabetes diastolic function was impaired and correlated with lower myocardial PCr/ATP ratios, while systolic function was preserved [10]. The myocardial PCr/ATP ratio reports on the steady-state balance between ATP turnover and ATP synthesis, and is as such an index of the in vivo myocardial energy status. It can be quantified non-invasively with phosphorus-31 magnetic resonance spectroscopy (^31^P‑MRS) [12].

### Mitochondrial dysfunction in HFpEF

Myocardial energy deficiency in HFpEF can be explained by mitochondrial dysfunction. Mitochondrial dysfunction in HFpEF is considered mainly to be a consequence of chronically increased oxidative stress, overexpression of proinflammatory cytokines, and other factors common in HFpEF known to be associated with mitochondrial abnormalities such as aging, renal dysfunction, and insulin resistance (Fig. 1, arrow 1) [7, 13, 14]. On the other hand, mitochondrial dysfunction is linked to insulin resistance, thus closing a first vicious circle (Fig. 1, arrow 4) [15, 16]. Notably, similar associations between mitochondrial dysfunction and hypertension or obesity have been observed [11, 17]. Importantly, this mechanism appears to be reversible: weight loss resulted in a near normalisation of the PCr/ATP ratio in parallel with improved diastolic function [17].

Another important contributing factor in HFpEF is the severely attenuated peak myocardial oxygen delivery during exercise, possibly related to endothelial dysfunction and impaired vasodilator capacity of the microcirculation [18]. When the energy supply-demand mismatch in heart failure further deteriorates mitochondrial function (by continuous oxidative stress), a second vicious circle is closed (Fig. 1, arrow 3) [7].

### Trimetazidine to improve mitochondrial efficiency

To test the ‘energy depletion’ hypothesis [9], we propose a novel intervention with a metabolism-modulating drug: trimetazidine. This drug has been approved worldwide for the symptomatic treatment of chronic stable angina [19]. Trimetazidine partially inhibits mitochondrial fatty acid β‑oxidation, by blocking long-chain 3‑ketoacyl-CoA thiolase, and concomitantly enhances glucose oxidation, because these two processes are tightly and inversely coupled by the Randle cycle [7]. As such, mitochondrial efficiency improves, because more ATP per mole of oxygen is produced via glucose oxidation. In addition, trimetazidine improves insulin sensitivity, which increases myocardial glucose uptake and enhances glucose oxidation [16]. Fragasso et al. reported that trimetazidine treatment for 3 months resulted in a 33% increase in the myocardial PCr/ATP ratio in HFrEF patients [20].

Multiple small randomised trials with trimetazidine in ischaemic and non-ischaemic cardiomyopathy have been performed in HFrEF patients: meta-analysis of these HFrEF trials demonstrates improvement in systolic and diastolic function resulting in a reduction in *N*-terminal pro-brain natriuretic peptide (NT-proBNP) as well as an improvement in symptoms, quality of life, and exercise tolerance after 3–6 months of treatment [19]. Studies with a follow-up of 2–5 years report a 10–50% reduction in hospitalisation and an absolute reduction in all-cause mortality of 11–30% [19]. Until now, trimetazidine or other metabolic modulators have not been tested for the treatment of HFpEF.

## Methods

This study will be conducted in accordance with the guidelines for Good Clinical Practice and with the Declaration of Helsinki. The study has been approved by the Medical Ethical Committee of VU University Medical Center, Amsterdam, The Netherlands (NTR: NL7830). Recruitment began in May 2019, and the study is expected to end in December 2020.

### Objectives

Our primary endpoint is to assess the effect of a 3-month trimetazidine treatment in patients with HFpEF on the change in exercise pulmonary capillary wedge pressure (PCWP) at specified exercise workloads.

Our key secondary endpoint is to evaluate the change in the myocardial PCr/ATP ratio measured by ^31^P‑MRS, and its relation to the primary endpoint. Additionally, we assess safety (frequency of adverse events) and explore NT-proBNP levels, C‑reactive protein level, 6‑min walk distance, and quality of life (Kansas City Cardiomyopathy Questionnaire, EQ-5D).

### Study design

**Fig. 2:**
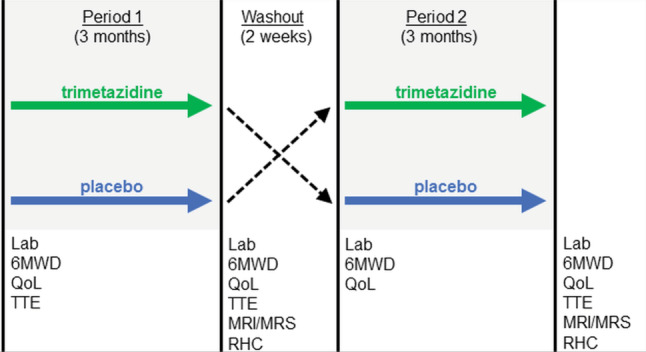
DoPING-HFpEF study design. A complete clinical assessment is scheduled at the end of each treatment period. *6MWD* 6-min walk distance, *QoL* quality of life, *TTE* transthoracic echocardiogram, *MRI/MRS* magnetic resonance imaging and magnetic resonance spectroscopy, *RHC* (exercise) right heart catheterisation

DoPING-HFpEF is a phase II (proof-of-principle) single-centre, double-blind, placebo-controlled, cross-over intervention study (Fig. 2). The study will include 25 HFpEF patients, recruited from the Amsterdam University Medical Centers’ dyspnoea/HFpEF outpatient clinic and network centres [21]. The duration of the study is two study periods of 3 months separated by a 2-week washout period (<7 months in total). Visits are planned at the start, after 6 weeks, and at the end of each treatment period.

### Study population

The DoPING-HFpEF study will enroll patients with the diagnosis and symptoms of HFpEF, without contra-indications for the study drug or procedures (see Tab. 1 for all inclusion and exclusion criteria).

**Table 1:** Inclusion and exclusion criteria

**Inclusion criteria:**
1. Diagnosis of HFpEF a. signs and/or symptoms of heart failure, NYHA II or higher (and ambulant) b. LVEF $\geq$50% (by any modality) c. evidence of LV diastolic dysfunction (one or more of the following): – PCWP at rest >15 mm Hg – PCWP during exercise $\geq$25 mm Hg [21] – diastolic dysfunction grade ≥II on echocardiogram with a NT-proBNP level >125 pg/ml d. no other significant cardiac (e.g. significant valvular disease or infiltrative cardiomyopathy) or extra-cardiac condition (e.g. severe COPD) that explains symptoms
2. Clinically stable (no change in diuretic therapy >1 month), co-morbidities managed
3. Informed consent
**Exclusion criteria:**
1. Current acute decompensated heart failure, requiring augmented therapy with intravenous diuretics, vasodilators, and/or inotropic drugs
2. Acute coronary syndrome, transient ischaemic attack/cerebrovascular accident, major surgery within the previous 3 months
3. Suspected major myocardial scarring (e.g. due to myocardial infarction)
4. Not able to undergo the complete study protocol (RHC, MRI, 6MWD)
5. Contra-indication for trimetazidine (severe kidney failure with an eGFR <30 ml/min/1.73 m^2^, parkinsonism or medication use that may cause parkinsonism)
6. Doubt about compliance
7. Pre-menopausal women who are nursing, pregnant, or of child-bearing potential and not practicing an acceptable method of birth control
8. Chronic absorption problems
9. Estimated life expectancy <1 year
10. Current or previous (<3 months) treatment with any SGLT‑2 inhibitor

*HFpEF* heart failure with preserved ejection fraction, *NYHA* New York Heart Association, *LVEF* left ventricular ejection fraction, *PCWP* pulmonary capillary wedge pressure, *NT-proBNP* *N*-terminal pro-brain natriuretic peptide, *COPD* chronic obstructive pulmonary disease, *RHC* right heart catheterisation, *MRI* magnetic resonance imaging, *6MWD* 6-min walk distance, *eGFR* estimated glomerular filtration rate, *SGLT‑2* sodium-glucose co-transporter 2

### Intervention: trimetazidine

Trimetazidine is administered orally by (encapsulated) tablets. Trimetazidine is contra-indicated in severe renal impairment (estimated glomerular filtration rate <30 ml/min/1.73 m^2^). It has a safe pharmacological profile: most important but rare side-effects are parkinsonism and restless legs syndrome. No important interactions have been reported and no safety concern has been observed in the elderly population.

The dosage of trimetazidine 20 mg three times daily (TID) (twice daily in the case of moderate renal impairment) was chosen for our current study for the following reasons: (1) clinically relevant metabolic effects on the heart have been demonstrated by ^31^P‑MRS and positron-emission tomography for this dosage in HFrEF patients [16, 20]; (2) the vast majority of the literature on trimetazidine studied this dosage, and more specifically all heart-failure-related studies have used either trimetazidine 20 mg TID or 35 mg modified release twice daily [19], allowing a direct comparison of our results with the literature; and (3) to achieve the highest efficacy we use the maximally advised dosage. Adherence is checked by pill counting and analysing plasma trimetazidine levels.

### Procedures

#### Exercise right heart catheterisation

Via a jugular venous approach, right heart catheterisation (RHC) is performed with a fluid-filled, balloon-tipped, flow-directed 7F Swan-Ganz catheter [21, 22]. Zero reference pressure level is set at the mid-axillary level. Subjects perform a stepped incremental bicycle ergometer test in a supine position, starting at 20 W and incrementing by 20 W every 3 min until exhaustion. PCWP during exercise is measured at the end of every stage, along with the standard haemodynamic parameters.

#### Magnetic resonance protocol

Magnetic resonance (MR) examinations are performed with subjects positioned supine in a 3 T MR system (Ingenia, Philips, Best, The Netherlands) equipped with vendor-supplied 16-element anterior and 12-element posterior coil arrays for proton (^1^H) signal reception, and include the following acquisitions: standard cine series of LV long-axis and short-axis views covering the whole heart; myocardial native T_1_ and T_2_ mapping, and localised ^1^H‑MRS of the septum. Next, localised ^31^P‑MRS of the left ventricle is performed using a 14-cm-diameter ^31^P surface coil positioned over the heart with the subject in a supine position, as described previously [12]. Left and right ventricular and atrial volumes, LV myocardial mass, and myocardial T_1_ and T_2_ are quantified using commercially available software packages. Stroke volume and LV ejection fraction will be calculated. Myocardial triglyceride and total creatine levels are estimated relative to the total water signal by fitting the ^1^H‑MR spectra in jMRUI [23]. The myocardial PCr/ATP ratio is estimated by calculating the ratio of the PCr and γ‑ATP signal amplitudes in the ^31^P‑MR spectra, corrected for partial saturation [12].

### Outcome, statistics and sample size calculation

The primary endpoint is change in exercise PCWP. Exercise PCWP standardised for a level of exercise is a novel primary endpoint in HFpEF trials that was introduced in the REDUCE-LAP HF trial [24]. This endpoint correlates with more traditional heart failure outcome measures, such as New York Heart Association (NYHA) class, exercise capacity, and heart-failure-related quality of life [25, 26].

The primary endpoint will be tested by a repeated measures ANOVA. However, a power calculation based on this statistical test would require imputation of multiple unknown variables, making it less reliable. Therefore, we simplified the power calculation and derived it from a paired *t*-test, which is also more conservative. Based on the data from the REDUCE-LAP study, we defined a clinical relevant mean reduction of exercise PCWP (at 20 W) of ∆ = 3.2 mm Hg with a standard deviation σ = 5.0 mm Hg [24]. Based on the results of the REDUCE LAP-HF I trial, showing only a minor change in exercise haemodynamics in the control group after 1 month in relatively unstable patients (NYHA III–IV) [24], we expect only a small intra-individual variation in exercise haemodynamics in our relatively stable NYHA II–III patient group. Power calculation (one-sided level of significance α = 0.05, power 1‑β = 0.80) for a paired comparison predicts a required sample size of *n* = 18 (NCSS PASS 15.0.5, Keysville, UT, USA). To compensate for potential drop-out, we increased the total number of patients for this study to *n* = 25.

Our key secondary endpoint, change in myocardial PCr/ATP, will be tested by a paired *t*-test. Fragasso et al. reported a change in the myocardial PCr/ATP ratio after trimetazidine treatment of (mean ± σ) 1.35 ± 0.33 to 1.80 ± 0.50 [20]. Based on these numbers, power calculation (one-sided, α = 0.05, 1‑β = 0.80, σ (difference) = 0.60) predicts a required sample size of *n* = 13, which is less than the total number of inclusions planned for this study.

## Discussion

The DoPING-HFpEF trial is a mechanistic study that aims to evaluate the relevance of mitochondrial dysfunction in HFpEF. The proposed intervention builds on the paradigm that has been proposed by Paulus et al., which assigns systemic inflammation and endothelial dysfunction a central role in the disease process of HFpEF, with oxidative stress and mitochondrial dysfunction as consequences [5]. Recently, the relevance of coronary microvascular disease and subclinical ischaemia in HFpEF was demonstrated [27, 28], but a direct mechanistic link of these observations with LV diastolic dysfunction remains unclear. We propose that mitochondrial dysfunction may be the missing link between systemic inflammation and diastolic dysfunction (Fig. 1). With trimetazidine, we have an attractive agent that directly affects mitochondrial function by improving its efficiency [19, 20], to test the ‘energy depletion’ hypothesis in HFpEF.

Almost all HFpEF-trials, including TOPCAT and PARAMOUNT, used high natriuretic peptide levels and echocardiographic evidence of LV dysfunction to define the study population [2]. We, and others, have reported that in HFpEF patients at an early but symptomatic stage (elevated PCWP at exercise only) natriuretic peptide levels are frequently normal, because LV wall tension is typically only mildly elevated and obesity indirectly lowers natriuretic peptide levels [21, 22]. Also, there is increasing awareness that echocardiographic parameters to assess LV diastolic function are not always accurate [29, 30]. We allow inclusion of patients based on haemodynamic criteria alone (the current gold standard for the diagnosis of HFpEF) with assessment of symptoms, without the necessity to exceed a certain natriuretic peptide threshold. As such, we expect to include a study population at a relative early stage of the disease, which is possibly more susceptible to therapy.

Because we primarily aim to improve LV diastolic function, we choose ‘change in exercise PCWP’ as our primary endpoint, which has been shown to correspond to conventional heart failure outcome measures [24–26]. Moreover, exercise RHC is the most sensitive method to detect therapeutic effects [22], thus allowing for sufficient statistical power despite the relatively small sample size of this proof-of-principle study. To confirm the mechanism of action of trimetazidine in our HFpEF patients and because improvement of myocardial mitochondrial efficiency is a central aspect in our hypothesis, we included ^31^P‑MRS in our study protocol. Localised ^31^P‑MRS allows for a non-invasive assessment of the myocardial energy status [12, 20], which will facilitate the differentiation between sufficient or insufficient restoration of myocardial energy metabolism to explain the effects of the trimetazidine intervention on the primary endpoint.

In conclusion, this study will test a novel approach in the treatment of HFpEF patients with trimetazidine, a metabolic drug with a favourable pharmacological profile that is readily available. Sophisticated measurements (exercise RHC and ^31^P‑MRS) will provide mechanistic insights. Ultimately, if the results of this trial are encouraging, data of this study will be used to design a large multi-centre, randomised clinical trial.
